# Translation, Cultural Adaptation, and Content Validity of the Saudi Sign Language Version of the General Nutrition Knowledge Questionnaire

**DOI:** 10.3390/nu16162664

**Published:** 2024-08-12

**Authors:** Jenan M. Aljubair, Dara Aldisi, Iman A. Bindayel, Madhawi M. Aldhwayan, Shaun Sabico, Tafany A. Alsaawi, Esraa Alghamdi, Mahmoud M. A. Abulmeaty

**Affiliations:** 1 Department of Community Health Sciences, College of Applied Medical Sciences, King Saud University, Riyadh 11362, Saudi Arabia; 442204508@student.ksu.edu.sa (J.M.A.); ebandael@ksu.edu.sa (I.A.B.); maldhwayan@ksu.edu.sa (M.M.A.); tafanyalsaawi@gmail.com (T.A.A.); esraa51421@gmail.com (E.A.); mabulmeaty@ksu.edu.sa (M.M.A.A.); 2 Biochemistry Department, College of Science, King Saud University, Riyadh 11451, Saudi Arabia; ssabico@ksu.edu.sa

**Keywords:** translation, cultural adaptation, content validity, general nutrition knowledge questioner, hearing impaired, sign language, WHO translation protocol

## Abstract

Profoundly hearing-impaired individuals lack health-promotion education on healthy lifestyles, and this may be due to communication barriers and limited awareness of available resources. Therefore, providing understandable healthy eating knowledge and a proper education evaluation via a questionnaire is vital. The present study aimed to translate, culturally adapt, and validate the content of a Saudi sign language version of the General Nutrition Knowledge Questionnaire (GNKQ). The study followed the World Health Organization guidelines for the translation and cultural adaptation of the GNKQ, using two-phase translation (from English into Arabic and then from Arabic into Saudi sign language), including forward-translation, back-translation, and pilot testing among profoundly hearing-impaired individuals. A total of 48 videos were recorded to present the GNKQ in Saudi sign language. The scale-level content validity index (S-CVI) value was equal to 0.96, and the item-level content validity index (I-CVI) value for all questions was between 1 and 0.9, except for question 6 in section 1, which was 0.6; this discrepancy was due to religious, social, and cultural traditions. The translation, cultural adaptation, and content validity of the Saudi sign language version of the GNKQ were satisfactory. Further studies are needed to validate other measurement properties of the present translated version of this questionnaire.

## 1. Introduction

According to the World Health Organization (WHO), over 5% of the world’s population has a hearing impairment. Additionally, it is estimated that one in every ten individuals will develop a hearing impairment, which is expected to double by 2050 [1]. Hearing impairment is defined as hearing loss > 35 decibels (dB) [1]. The prevalence of hearing impairment increases with age above 60 years [1]. The hearing impairment community in Saudi Arabia exceeds 720,000 individuals, according to the latest statistics from the General Authority of Statistics (2017), with the highest number found in the capital, Riyadh [2]. The number of individuals with mild, intermediate, and profound hearing impairments was reported to be 65,752, 11,346, and 2984, respectively [2].

Sign language is the primary communication method for profoundly hearing-impaired individuals [3]. It is defined as “visual language used for hand movements and facial expressions by people with speech and hearing disabilities to communicate” [3]. With a profound hearing impairment, individuals find it challenging to communicate. Sign language-recognition systems translate signs into natural languages, decreasing the gap between individuals with a profound hearing impairment and individuals with normal hearing [3]. In Saudi Arabia, individuals with a profound hearing impairment use Saudi sign language, which is different from standard Arabic [3]. Thus, it is not possible to generalize this version to other varieties of Arabic (e.g., Levantine or Egyptian).

At the individual level, a hearing impairment impacts many aspects of life, including communication and speech, cognition, social isolation and loneliness, depression, education, and knowledge [4]. Due to the lack of health-promotion education on healthy eating and regular exercise for the hearing-impaired population, especially among elderly members, both overweight and obesity have been reported [5]. Among the attributed factors are an excess intake of calories from unsaturated fats, oils, and sugar-containing beverages; a lack of physical activity and understanding of its importance to health; social isolation; and loneliness [4,6,7]. A cross-sectional study was conducted at the University of Hail, Saudi Arabia, between September 2010 and September 2011, with 65 normal-hearing (−10 to 15 dB) and 42 hearing-impaired female (greater than 16 dB) students aged 18–21 [8]. A food frequency questionnaire was used to evaluate food intake, and vitamin levels were measured for each food and compared with the recommended daily allowance (RDA) for both groups. The consumption levels of all vitamins, except for vitamins D and B9, met the RDA in the normal-hearing group. The consumption levels of all vitamins in the hearing-impaired group were higher than the RDA and those of the normal-hearing group [8].

The General Nutrition Knowledge Questionnaire (GNKQ) was developed in the early 1990s in the United Kingdom (UK) [5]. The first validation was in 1999, which aimed to develop a reliable and valid GNKQ to provide a comprehensive measurement of the nutritional knowledge of UK individuals. The GNKQ was reviewed by four dieticians and four psychologists to select the best questions in terms of the accuracy of dietary knowledge and clarity; the internal consistency of the questionnaire items was good (Cronbach’s alpha = 0.70–0.97), and the test–retest reliability was >0.7 [5]. The second validation of the GNKQ revision was carried out in 2016, and the aim was to update the GNKQ to include current nutritional advice. The GNKQ was revised by an expert panel composed of 2 dieticians, 15 dietetics students, and 3 health psychologists to assess the nutritional knowledge and level of difficulty. It reported a high reliability (Cronbach’s alpha = 0.93) [9].

Due to the lack of health-promotion education on healthy eating, the intake of unhealthy food among profoundly hearing-impaired individuals is high [10]. It is speculated that this lack of health-promotion education is related to the lack of culturally adequate tools. Currently, there is no Saudi sign language or Arabic sign language version of the GNKQ questionnaire. Hence, the present study aims to translate and validate the content of the GNKQ into a Saudi sign language tool, using the WHO guidelines, to aid in adequately assessing the dietary and lifestyle aspects of individuals with profound hearing impairments in both community and research settings.

## 2. Materials and Methods

This study was conducted to translate the GNKQ into Saudi sign language using the WHO guidelines for translation, adaptation, and validation as a standardized translation protocol [11]. This translation protocol was developed to achieve various language versions of an instrument that are conceptually equal when applied to various cultures [11]. As there was no Saudi sign language or Arabic language version of the GNKQ questionnaire, permission was obtained from the original authors of the questionnaire [9] and publication sources (Spring Nature). This protocol was approved by the Institutional Review Board (IRB) of King Saud University (IRB No.: KSU-HE-23-934). Written informed consent was obtained from each participant. This study took place from June 2023 to March 2024.

### 2.1. The GNKQ Tool

The original version of GNKQ questionnaire is divided into five sections: section 1 has 9 questions about what advice is expected from experts regarding nutrition; section 2 has 10 questions about the awareness of food groups and the nutrients that they contain; section 3 has 13 questions about the choice of foods and food labeling; section 4 has 16 questions about health problems or diseases related to diet and weight management; and, finally, section 5 has 10 questions about personal information, including ethnic origin, marital status, and education level.

### 2.2. Translation Process

#### 2.2.1. Translation from English into Arabic

The first step (forward-translation) involved translating the original GNKQ from English into Arabic as the first target language, which was carried out by three bilingual experts in nutrition. All the experts were native Arabic speakers fluent in English. The translation was based on achieving the linguistic and conceptual meaning of the original English version rather than producing a word-for-word translation. A fourth bilingual academic professor in nutrition compared the three forward-translated versions of the GNKQ regarding the ambiguities of words, sentences, and meanings to produce a single translated version.

The second step (back-translation) involved translating the Arabic version of the GNKQ into English, which was carried out by a bilingual expert in medical translation who is native to English, is fluent in Arabic, and has experience in the medical field. To ensure a high-quality back-translation, it was critical to check the accuracy of the initial Arabic version versus the original questionnaire and to choose well-qualified translators. The bilingual translator was blind to the original English version. Consequently, the research group reviewed the back-translation and applied the necessary changes to finalize the questionnaire.

Content validity was assessed by nine experts in nutrition (six academic associate professors in clinical nutrition and three clinical dietitians). Expert panels were invited to participate by email to evaluate and assist with the cultural adaptation of the questionnaire. After providing consent, the experts received an online link using Google Forms for the questionnaire. The online survey contained all 49 questions translated into Arabic. The expert panel then assessed each question on the following Likert scale for content-related validity: (A) not applicable, (B) unable to determine relevance, (C) relevant but required minor revisions, and (D) very relevant and succinct for clarity. A section was provided for suggestions on how to enhance the conceptual equivalency of the questionnaire when applicable. The responses obtained from the experts were used to calculate the mean score for the evaluation of the content validity and clarity of each question.

#### 2.2.2. Translation from Arabic into Saudi Sign Language

The translation of the tool from Arabic into Saudi sign language (the second target language) as a video clip was carried out by an independent bilingual translator whose mother language is Arabic and who is fluent in Saudi sign language and certified as a sign language translator by the Arabic Federation of the Deaf Organization. Even with the limited vocabulary of the Saudi sign language, the translation was based on achieving the conceptual meaning of the Arabic version rather than producing a word-for-word translation. Each question had a video clip between 1 and 2 min long in Saudi sign language, with a black background and picture for more clarity, and a total of 48 video clips were produced. After that, the second translated questionnaire was considered ready for the second validation.

A process was approved to assess the content validity and determine the conceptual and content equivalence of the Saudi sign language video version. This process involved two expert investigators in Saudi sign language; the first was a professor in the education track of hearing impairment, who was certified as a sign language translator by the Arabic Federation for Organizations of the Deaf, while the second was an expert translator in Saudi sign language, certified as a sign language translator by the Saudi Sign Language Association.

Two experts were invited to participate by email to evaluate and assist with the videos via an online link using Google Forms. The videos contained 49 Saudi sign language items of the questions of the GNKQ. Then, the experts assessed each question on a Likert scale for content-related validity, with the Likert scale being the same as that used for the first evaluation of content validity. A section was provided for suggestions on how to enhance the conceptual equivalency of the video when applicable. The responses obtained from the experts were used to calculate the mean score for the evaluation of the content validity and clarity of each video. Based on the results of this stage, the last version of the questionnaire and videos was revised into a final version, which was ready for the following step: pilot testing on Saudi adults with profound hearing impairments, both males and females, who used Saudi sign language.

### 2.3. Pilot Testing of the Pre-Final Version

**Figure 1:**
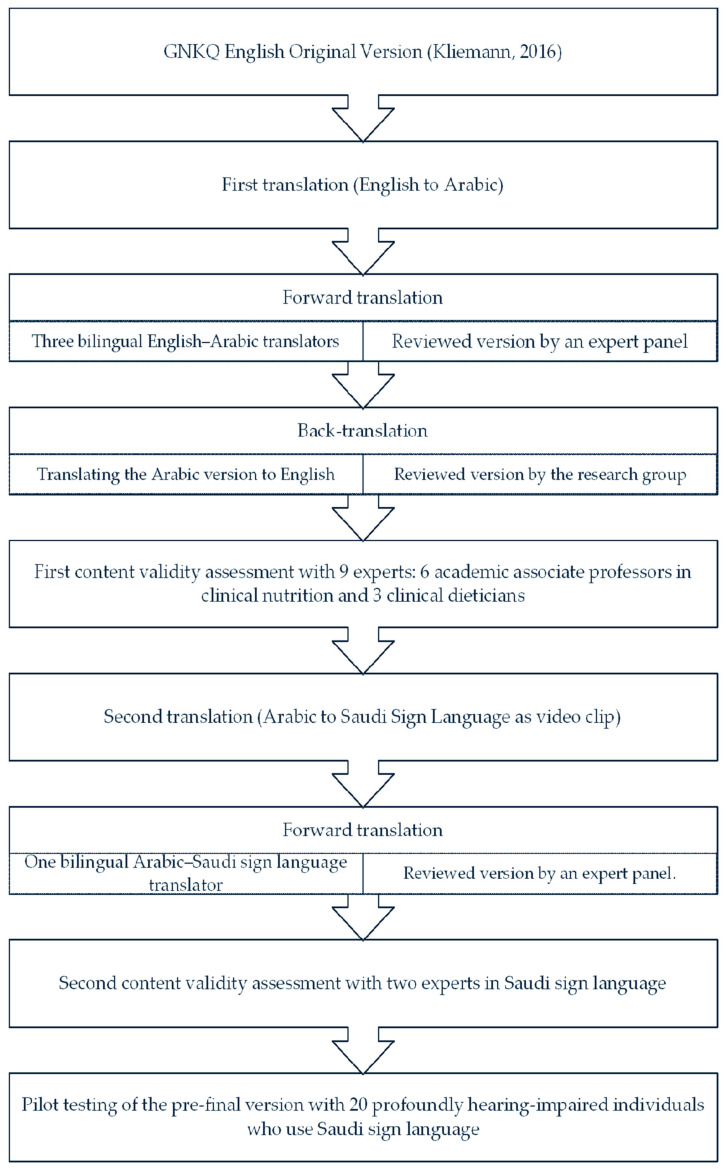
Process stages of translation of the Saudi sign language version of the General Nutrition Knowledge Questionnaire [9].

A total of 20 Saudi individuals, 14 females and 6 males, with profound hearing impairments (defined as 90 dB or above [12]) participated in this pilot test by completing the pre-final video version of the questionnaire via a face-to-face interview. After completing the sign language video questionnaire, the participants were asked whether the video-translated questions were 1 = clear and understandable, 2 = difficult to answer, 3 = confusing to answer, and 4 = relevant to the topic using a Likert scale (1–4); this scale was adopted from [13,14]. Furthermore, the participants were requested to suggest changes to enhance and clarify the Saudi sign language video questionnaire version. The interviewer was requested to identify any video questions with an unclear meaning, and, when appropriate, suggestions were made to improve the questionnaire’s understanding, clarity, and cultural adaptation. The stages of the GNKQ translation process are summarized in Figure 1.

### 2.4. Cultural Adaptation

Cultural adaptation was carried out in every step of the translation process, during which the wording of some items was also changed to better fit the Saudi background, culture, and religion without significantly changing meaning so that the translated version still matched the original GNKQ.

### 2.5. Statistical Analysis

The categorically measured variables are described using percentages. The scale validity index (S-CVI) and content validity index (I-CVI) were used, with an I-CVI of 0.78 or higher and an S-CVA/Ave of 0.90 or higher representing the minimum acceptable indices. The calculation of the scale validity and content validity indices was conducted according to Yusoff (2019) [15]. The calculation process of the I-CVI is the number of experts in agreement divided by the number of experts (agreed on item/number of experts), while that of the S-CVA/Ave is the average of the I-CVI scores across all items [15].

## 3. Results

### 3.1. Translation from English into Arabic (Forward-Translation)

The English version of the questionnaire was first translated into Arabic. With regard to section 1 question 1, the expert panel suggested updating the number of fruit and vegetables to 5 servings/day and 6 to 8 cups of water/day according to guidelines [16]. Additionally, they recommended adding the word “fruit” following the number in question 2 for clarification. The panel recommended providing an example of the types of fat in question 3 and adding the word “serving” in question 8 for clarification.

In section 2, question 1, the expert panel suggested changing “diet cola drink” to soft drink without white sugar, diet, or lite cola drink to aid clarification. In question 6, the change of “Poly and monounsaturated” to “unsaturated” was recommended for simplicity. The addition of a definition of trans fat in question 7 and an example of processed foods in question 9 was recommended to clarify the meaning.

In section 3, question 3, the expert panel suggested explaining “tartar sauce” and providing the sauce’s ingredients. In addition, providing an explanation for the “Traffic lights on nutrition labeling” in question 10 was suggested to aid clarification. Furthermore, determining the type of fruit and vegetable in the multiple-choice answer in question 8 and changing the type of bread to whole grain bread instead of “bread only” in question 11 were recommended to aid clarity. Finally, it was suggested that adequate sleep and physical activity be added as options that can help individuals maintain a healthy weight in question 13, according to the current recommendation by the Healthy Weight, Nutrition, and Physical Activity [17].

For cultural adaptation, in section 1, question 5, the panel recommended the change of “Salmon” to “Fish”. In section 2, question 1, the expert panel suggested changing “natural yogurt” to “yogurt without additives”. Furthermore, it was recommended that “Plantains” in section 2 question 5, an unknown fruit in Saudi culture, be removed. In section 4, it was suggested that the word “Salmon” be changed to “Fish” for cultural adaptation. The questions in section 5 were about the participants’ background characteristics regarding cultural background and ethnicity. Some personal questions considered inappropriate for use in Saudi culture were removed in the forward-translation.

### 3.2. Back-Translation from English to Arabic

In section 1, question 1, the researcher group suggested changing the translated word “expert” to “health expert”. In question 2, the researcher group suggested changing the translated phrase “mainly daily rations” to “minimum daily portion” according to the original version of the GNKQ. In section 3, question 2, the researcher group suggested changing the translated phrase “fungus” as a food ingredient to “Mushroom” according to the original version of the GNKQ. In section 3, question 3, the researcher group suggested changing the translated phrase “puree” to “puree potato” and the translated phrase “grilled sweet potatoes” to “roasted potato” according to the original version of the GNKQ. In the same section, in question 5, the researcher group suggested changing the translated phrase “Raspberry confectionery” to “Berry Sorbitet” according to the original version of the GNKQ. Finally, in the same section, question 11, the researcher group suggested changing the translated phrase “dietary foods” to “diet food” according to the original version of the GNKQ.

### 3.3. Translation from Arabic into Saudi Sign Language (Forward-Translation)

The Arabic version was translated into Saudi sign language. A deaf photographer recorded 48 video clips one to two minutes long, following technical recommendations for camera format and lighting, featuring a translator wearing solid-colored clothing that contrasted with their skin tone and the background, in accordance with international guidelines designed to ensure the clear interpretation of sign languages [18] (Supplementary Material). In response to section 1, the expert panel suggested changing the words of section 1, question 1, from “Do health experts recommend that people should be eating more, the same amount, or less of the following foods?” to “Do you think that nutrition experts advise individuals to eat each of the following foods or to stay away from them as much as possible?” to improve comprehension and clarity for profoundly hearing-impaired individuals. In section 2, question 2, the expert suggested adding the word “per day” at the end of the question, followed by the number of fruits in the answer for clarification. Additionally, the expert panel suggested changing the location of the answer option “not sure” to the middle between another two-answer option and adding a numerical one in each option to avoid conflict among profoundly hearing-impaired individuals in all multiple-answer questions. Finally, 25 out of 50 items (50%) were modified in terms of sentence structure and alternative wording in both phases of translation.

### 3.4. Pilot Testing of the Pre-Final Version among Profoundly Hearing-Impaired Volunteers

The participants suggested clarifying the meaning of carbohydrates, protein, cholesterol, calcium, sodium, antioxidants, and fiber by adding pictures to section 1 (question 9), section 2 (questions 2, 3, 4, 5, and 8), section 3 (question 6), and section 4 (questions 1, 3, and 7), as well as changing “baked beans” to “canned beans” as a cultural adaptation in section 2 (questions 2 and 4). Additionally, in section 1 (question 8), the participants did not fully understand the meaning of “serving” as a word. They suggested adding clarification that a serving is a portion of fruit in both the question and answer.

### 3.5. Descriptive Analysis

Table 1 shows the descriptive results for the sociodemographic characteristics of the pilot sample (*n* = 20). More than half were between 30 and 39 years old, and the female participants represented 70% of the sample. Moreover, 75% of the participants held either a diploma or a bachelor’s degree. Finally, 70% of the participants were employed. 

### 3.6. Content Validity

The mean grades for the evaluation of the content validity, understandability, and clarity of each item and semantic differentiation were calculated using the responses obtained from the experts. In order to calculate the CVI, the relevance rating was recorded as 1 (with a relevance scale of C—relevant but requires minor revision or D—very relevant) or 0 (with a relevance scale of A—not applicable or B—unable to determine relevance) [15]. The S-CVI (scale-level content validity index) was calculated and found to be equal to 0.96. As shown in Table 2, the value of the I-CVI for all questions was between 1 and 0.9, except for question 6 in section 1, which was 0.55; this discrepancy was due to religious, social, and cultural traditions. Most items had a universal agreement (UA), meaning that 100% of the experts agreed. The S-CVI/Ave (scale-level content validity index based on the average method) was equal to 0.96, and the S-CVI/UA (scale-level content validity index based on the universal agreement method) was equal to 0.76 (Table 3).

## 4. Discussion

This study developed the first Saudi sign language version of the General Nutrition Knowledge Questionnaire by following the WHO’s translation process with experts in sign language and health [11]. The aim was to translate the Saudi sign language of the General Nutrition Knowledge Questionnaire and to adapt it for use in Saudi culture. The GNKQ, which was developed by Parmenter and Wardle [5] and updated by Kliemann [9], includes the following sections: dietary recommendations by experts, food groups, healthy food choices, diet, weight management, and diseases. The original version of the questionnaire was in English [5]. However, after translating it into Arabic and using Saudi sign language in a video version in this study, fewer common dishes/foods were replaced with more familiar foods in Saudi eating cuisine/patterns. This was similar to another published study [19], which aimed to determine the validity and reliability of a GNKQ for Romanian adults; the researchers replaced fewer common foods/dishes with similar, more common foods in Romanian eating cuisine/patterns in the GNKQ.

Most of the translation problems encountered in this study were due to unclear meanings, multiple meanings, compound words, grammatical problems, or rhetorical meanings. Other published studies have also encountered problems in translating tools from English to Brazilian Portuguese [20]. A personality disorder tool was translated from English to Brazilian Portuguese, and the researchers found that 60.2% of the items (56 out of 83 items) were modified [20]. Compared with our results, many items were modified, including sentence structure and alternative wording, with around 25 out of 50 items (50%) being modified in both phases of translation. Another study also translated a standardized questionnaire from English to Norwegian to address the same issue [14]. Another challenge encountered during the conduction of this study was the failure to understand individual words due to the scarcity of words in Saudi sign language, especially medical terminology.

In contrast, some participants understood the sentence but had difficulty in providing a score due to a lack of nutrition knowledge. Interestingly, the word “portion” was difficult to understand among the profoundly hearing-impaired individuals, both educated and non-educated, due to a lack of nutrition knowledge; thus, adding a definition of the term “portion”, as well as a cultural term, resulted in the use of “piece” as a synonym because this was more understandable. Compared with other published studies on the Brazilian translation, the word “hostile” was challenging for individuals with a low education level in Brazil to understand. Thus, a cultural term with a suitable phrase in the Brazilian language was adopted to be more understandable [20].

The participants in the pilot testing of the pre-final version of this study were profoundly hearing-impaired, belonging to the deaf community, and they represented different genders, ages, and levels of education. During the two phases of the translation process, close attention was paid to ensuring that the translated version (either the first phase of translation into Arabic or the second phase of translation into Saudi sign language) matched the original version of the GNKQ to ensure validity. In the video-based Saudi sign language version of the General Nutrition Knowledge Questionnaire, pictures were added for clarification, comparable to a published study [10], which added a picture in the GNKQ in English sign language. Furthermore, the translator wore solid-colored clothing that contrasted with the background when recording the video to ensure the clear interpretation of the sign language. This is comparable to a published study that used plain clothing that contrasted with the background when recording an oral health hygiene questionnaire in a video format for hearing-impaired adults in Saudi Arabia [21]. 

Regarding the content validation, it is worth highlighting that it is a prerequisite for any valid questionnaire and, thus, should be given the highest priority during the development process of any new questionnaire [22]. The I-CVI values for all questions were between 1 and 0.9, except for question 6 in section 1, which was related to alcohol consumption; the I-CVI for this question was 0.6 due to religious, social, and cultural traditions. Thus, this question was removed, comparable to in a previously published study that aimed to translate and validate an Arabic version of the Eating Behavior After Bariatric Surgery (EBBS) Questionnaire; the one item related to alcohol consumption was removed due to religious, social, and cultural traditions and, thus, low variance [13]. Most of the items in this study had a universal agreement: the S-CVI/Ave was equal to 0.96, and the S-CVI/UA was equal to 0.76. Therefore, based on the above calculation, it was concluded that the I-CVI, UA, S-CVI/Ave, and S-CVI/UA reached satisfactory levels, and, thus, the scale of the questionnaire achieved a satisfactory level of content validity comparable to that in a previously published study [15]. Another study aimed to translate, validate, and culturally adapt the English System Usability Scale questionnaire into Malay, Malaysia’s native language. The researchers found that the CVI was 0.91, indicating that it is a valid and reliable tool for assessing the serviceability of mobile apps in Malaysia [23].

### Limitations and Strengths

Some of the limitations of this study include the number of females in the pilot study being higher than the number of males and the females being more knowledgeable in nutrition than males, as well as the one-way translation in phase two (from Arabic into Saudi sign language), without back-translation (Saudi sign language into Arabic). In the second content validity assessment, the process was carried out by two experts in sign language due to the challenges of finding an expert certified as a Saudi sign language translator. Besides the limited vocabulary of Saudi sign language, the translation was based on achieving the conceptual meaning of the Arabic version rather than producing a word-for-word translation that could lead to an imprecise translation; to this end, we added pictures in the videos for more clarification. Nevertheless, this is the first Saudi sign language version of the GNKQ developed by following the WHO’s translation process with experts in sign language and health.

## 5. Conclusions

The translation, cultural adaptation, and content validity of the Saudi sign language version of the GNKQ, comprising 48 items, achieved satisfactory levels. The Saudi sign language version of the questionnaire can be used to estimate the level of nutrition knowledge among profoundly hearing-impaired individuals. This can lead to improving the nutritional status and increasing the awareness and knowledge of disease prevention in this population. Health risks can be prevented by addressing the social determinants of health, optimal health, and healthcare for the population and individuals with chronic disease. However, it is necessary to further confirm the validity and reliability of this questionnaire. It is suggested that the validity of the video questionnaire be officially completed by applying it in a large sample of profoundly hearing-impaired individuals in Saudi Arabia.

## Figures and Tables

**Table 1:** Sociodemographic characteristics of the Saudi participants with a profound hearing impairment (*n* = 20).

Gender	Male	6 (30)
	Female	14 (70)
Age	19–29	4 (20)
	30–39	11(55)
	40–49	4 (15)
	50–59	1 (5)
	60	1 (5)
Education level	Elementary	-
	Intermediate	1 (5)
	High school	4 (20)
	Diploma	7 (35)
	Bachelor’s	8 (40)
Occupation	Students	2 (10)
	Employed	15 (70)
	Unemployed	1 (5)
	Retired	2 (10)

**Table 2:** Content validity index (CVI) of each item.

No. Items	CVI
section 1	
1. Do you believe that experts recommend people to have more quantity, the same quantity, or less quantity of the following foods?	1
2. What are the main daily portions of fruits and vegetables recommended by experts? (One portion can contain, for example, an apple or hand grip of cut carrots)?	0.9
3. Which of those types of fats are recommended by experts to be eaten less?	1
4. Which type of dietary products is recommended by experts?	1
5. How many times per week are recommended by experts to have fatty fish (such as salmon)?	1
6. Approximately, how many alcoholic drinks is the maximum recommended per day (The exact number depends on the size and strength of the drink)?	0.55
7. How many times per week do experts recommend having breakfast?	1
8. If two fruit juice cups per day are consumed, how many daily rations of fruits and vegetables shall be calculated?	0.9
9. According to the “good eating” guide, how must the individual’s system of starches be? * (Guide that indicates the rates of food groups that an individual must have to get a balanced and healthy dietary system)?	0.9
section 2	
1. Do you believe that those foods and beverages usually contain added sugar in high or low rates?	0.9
2. Do you believe that those foods are usually high or low in salts?	1
3. Do you believe that those foods are usually high or low in fibers?	1
4. Do you believe that those foods represent a good source of protein?	1
5. Which of those foods do you believe that experts classified within the starchy group?	0.9
6. What is the main type of fats in each of those foods?	1
7. Which of those foods contain higher quantity of trans-fats?	0.9
8. Amount of calcium in cup of full cream milk as compared to cup of skimmed milk is:…	1
9. Which of the following food items contain more calories for the same quantity of the following foods…	1
10. Compared to unprocessed foods, processed foods contain:…	0.9
section 3	
1. If yoghurt is purchased from supermarket, which types contains a lower quantity of sugar?	1
2. If a person wants to drink soup from a restaurant, which of the following options contains the lowest amount of fats?	1
3. What is the healthy and balanced food option of the main course chosen from a restaurant?	1
4. Which of the following sandwiches is the healthy option as a meal?	1
5. What is the healthiest option of a dessert dish?	1
6. Which of the following groups of vegetables in salad provide the highest diversity of vitamins and antioxidants?	1
7. If a person wants to reduce the quantity of fats in his diet, but doesn’t want to leave the fried chips, which of the following foods will be the best choice?	0.9
8. One of the healthy methods of adding flavor to food without addition of more fats or salt is addition of:…	1
9. Which of the following methods of cooking require the addition of fats?	1
10. Traffic signs are often used on food labels; what does the yellow of fact content mean in food?	1
11. Light foods or diet foods are often considered good options because they are low in calories.	1
12. Looking at the first and second products, which of them contains the highest number of calories (kilocalories) per 100 g?	1
13. Looking at the first product, what are the sources of sugar on the list of ingredients?	1
section 4	
1. Which of the following diseases is related to low eating of fiber?	0.9
2. Which of the following diseases is related to the quantity of sugar eaten by the individual?	1
3. Which of the following diseases is related to the quantity of salt (or sodium) eaten by the individual?	1
4. Which of the following advice is given by experts for the reduction of the chances of cancer?	1
5. Which of the following advice is given by experts for the prevention of heart diseases?	1
6. Which of the following advice is given by experts for prevention of diabetes?	1
7. Which of the following foods are likely to increase the percentage of blood cholesterol?	1
8. Which of the flood foods is classified as containing a high glycemic index? * (Glycemic index is the measurement of food’s effect on the level of blood sugar, so an increase in glycemic index means a higher increase in blood sugar after food).	1
9. To keep a healthy weight, you must completely prevent fats.	1
10. To keep a healthy weight, have a high-protein diet.	1
11. Eating bread/rice food always causes overweight.	1
12. Eating food that contains fiber reduces likely overweight.	1
13. Which of the following options can help people keep a healthy weight?	1
14. If the Body Mass Index (BMI) of the individual is 23 kg/m^2^, what is his weight status?	0.9
15. If the Body Mass Index (BMI) of the individual is 31 kg/m^2^, what is his weight status?	1
16. Which of the following body shapes is related to increased cardiovascular diseases *?	0.9

* represents (Cardiovascular disease is a term that describes cardiovascular diseases, such as angina, heart seizure, heart failure, congenital heart diseases, and brain strokes).

**Table 3:** Relevance of the GNKQ items: expert ratings and agreement.

	Expert 1	Expert 2	Expert 3	Expert 4	Expert 5	Expert 6	Expert 7	Expert 8	Expert 9	Expert10	Expert 11		Expert Agreement	* I-CVI	**** UA
Items															
Q1-1	1	1	1	1	1	1	1	1	1	1	1		11	1	1
Q1-2	0	1	1	1	1	1	1	1	1	1	1		10	0.9	0
Q1-3	1	1	1	1	1	1	1	1	1	1	1		11	1	1
Q1-4	1	1	1	1	1	1	1	1	1	1	1		11	1	1
Q1-5	1	1	1	1	1	1	1	1	1	1	1		11	1	1
Q1-6	1	1	0	0	1	0	0	1	0	1	1		6	0.55	0
Q1-7	1	1	1	1	1	1	1	1	1	1	1		11	1	1
Q1-8	0	1	1	1	1	1	1	1	1	1	1		10	0.9	0
Q1-9	1	1	1	0	1	1	1	1	1	1	1		10	0.9	0
Q2-1	1	1	1	1	1	1	1	1	0	1	1		10	0.9	0
Q2-2	1	1	1	1	1	1	1	1	1	1	1		11	1	1
Q2-3	1	1	1	1	1	1	1	1	1	1	1		11	1	1
Q2-4	1	1	1	1	1	1	1	1	1	1	1		11	1	1
Q2-5	1	1	1	1	1	1	1	0	1	1	1		10	0.9	0
Q2-6	1	1	1	1	1	1	1	1	1	1	1		11	1	1
Q2-7	0	1	1	1	1	1	1	1	1	1	1		10	0.9	0
Q2-8	1	1	1	1	1	1	1	1	1	1	1		11	1	1
Q2-9	1	1	1	1	1	1	1	1	1	1	1		11	1	1
Q2-10	1	1	1	1	1	1	0	1	1	1	1		10	0.9	0
Q3-1	1	1	1	1	1	1	1	1	1	1	1		11	1	1
Q3-2	1	1	1	1	1	1	1	1	1	1	1		11	1	1
Q3-3	1	1	1	1	1	1	1	1	1	1	1		11	1	1
Q3-4	1	1	1	1	1	1	1	1	1	1	1		11	1	1
Q3-5	1	1	1	1	1	1	1	1	1	1	1		11	1	1
Q3-6	1	1	1	1	1	1	1	1	1	1	1		11	1	1
Q3-7	1	1	1	1	1	1	0	1	1	1	1		10	0.9	0
Q3-8	1	1	1	1	1	1	1	1	1	1	1		11	1	1
Q3-9	1	1	1	1	1	1	1	1	1	1	1		11	1	1
Q3-10	1	1	1	1	1	1	1	1	1	1	1		11	1	1
Q3-11	1	1	1	1	1	1	1	1	1	1	1		11	1	1
Q3-12	1	1	1	1	1	1	1	1	1	1	1		11	1	1
Q3-13	1	1	1	1	1	1	1	1	1	1	1		11	1	1
Q4-1	1	1	1	1	1	1	0	1	1	1	1		10	0.9	0
Q4-2	1	1	1	1	1	1	1	1	1	1	1		11	1	1
Q4-3	1	1	1	1	1	1	1	1	1	1	1		11	1	1
Q4-4	1	1	1	1	1	1	1	1	1	1	1		11	1	1
Q4-5	1	1	1	1	1	1	1	1	1	1	1		11	1	1
Q4-6	1	1	1	1	1	1	1	1	1	1	1		11	1	1
Q4-7	1	1	1	1	1	1	1	1	1	1	1		11	1	1
Q4-8	1	1	1	1	1	1	1	1	1	1	1		11	1	1
Q4-9	1	1	1	1	1	1	1	1	1	1	1		11	1	1
Q4-10	1	1	1	1	1	1	1	1	1	1	1		11	1	1
Q4-11	1	1	1	1	1	1	1	1	1	1	1		11	1	1
Q4-12	1	1	1	1	1	1	1	1	1	1	1		11	1	1
Q4-13	1	1	1	1	1	1	1	1	1	1	1		11	1	1
Q4-14	1	1	1	1	0	1	1	1	1	1	1		10	0.9	0
Q4-15	1	1	1	1	1	1	1	1	1	1	1		11	1	1
Q4-16	1	1	1	1	1	1	0	1	1	1	1		10	0.9	0
													** S-CVIlAve	0.96	
Proportion relevance	0.95	1	0.97	0.97	0.97	0.97	0.89	0.97	0.95	1	1		*** S-CVIlUA		0.76
	Average proportion of items judged as relevant among the eleven experts											0.96			

Note: * I-CVI, item-level content validity index; ** S-CVI/Ave, scale-level content validity index based on the average method; *** S-CVI/UA, scale-level content validity index based on the universal agreement method. Formula: * I-CVI = (agreed item)/(number of experts); ** S-CVI/Ave = (sum of I-CVI scores)/(number of items); *** S-CVI/UA = (sum of UA scores)/(number of items); **** universal agreement (UA): score “1” was assigned to items that achieved 100% agreement among experts.

## Data Availability

The original contributions presented in the study are included in the article/Supplementary Material. The corresponding authors can be contacted.

## Supplementary Materials

The online version contains supplementary material available at: https://docs.google.com/forms/d/1A4v8EIGoLInz0HHj-zNwBuf6nBMabpthB0g3stO5ciE/edit.
